# Experiencing more meaningful coincidences is associated with more real-life creativity? Insights from three empirical studies

**DOI:** 10.1371/journal.pone.0300121

**Published:** 2024-05-24

**Authors:** Christian Rominger, Andreas Fink, Corinna M. Perchtold-Stefan

**Affiliations:** Department of Psychology, University of Graz, Graz, Austria; IPCAS: Institute of Psychology Chinese Academy of Sciences, CHINA

## Abstract

Literature suggests a link between creativity and the perception of meaningful patterns in random arrangements, which is coined apophenia, patternicity, synchronicity, or the experience of meaningful coincidences. However, empirical research did not establish a clear link between real-life creativity and the experience of meaningful coincidences. In this three-study approach, we consistently found a connection between the experience of meaningful coincidences and creative activities as well as creative achievements. However, we did not obtain a consistent link with openness to experience or with peoples’ creative potential. By applying an internet daily diary approach, we found that the experience of meaningful coincidences fluctuates from day to day and that the number of perceived coincidences is associated with positive and negative affect. A third preregistered study showed that positive and negative affect might not serve as a strong mechanism that mediates the link between meaningful coincidences and real-life creative activities. We need further research to explore the reason for this robust link between meaningful coincidences and real-life creativity.

## Introduction

The experience of meaningful coincidences is defined as the sudden perception of a tight and significant connection of events which are, however, objectively unrelated, such as thinking about a friend and receiving a phone call from exactly this friend [1]. In these moments, we believe to experience that an inner event causes an outer event, and that thinking about the friend might have led to the call of the friend. This elicits a strong feeling that somehow, both events are related and take place in synchrony. People, show interindividual differences in the number of these and similar experiences [2]. A higher frequency of meaningful coincidences is associated with paranormal beliefs and positive schizotypy [3,4], but at the same time presents its own, distinct characteristics [2,5]. While linked to positive schizotypy and at the extreme, to psychiatric illness of schizophrenia, the experience of illusory connections and meaningful patterns between unrelated events, semantic concepts, and random arrangements (i.e., apophenia, [6]; but see [7] for a critical discussion of pareidolias as a surrogate for visual hallucinations) might also represent a benevolent and useful human trait, which is relevant for creativity [2,8–10].

Creativity can be understood as a potential (i.e., creative potential), a personality trait (i.e., openness), an activity (i.e., creative activities), as well as an achievement (i.e., creative achievements; see e.g., [11,12]). The production of unique and original ideas, which are useful is at the core of all facets of creativity ([13]; but see e.g., [14,15] for an ongoing debate). Following this definition, seeing connections and meaningful patterns which are *hidden from the view of others (cf*. [16]) might help to overcome mental blocks, enhance thinking out of the box, and increase the chance to come up with more creative ideas. Therefore, the propensity to experience meaningful coincidences might represent a personality trait associated with a higher creative potential. In line with this, a meta-analysis indicated a positive relationship between creativity and positive aspects of schizotypy [17]. More specifically, several authors suggested a link between meaningful coincidences, and related phenomena such as synchronicity, patternicity, and apophenia with creativity [2,10,18,19]. However, there is only little direct empirical evidence available indicating a connection between creativity and the propensity to experience meaningful coincidences.

What has been done so far? Rominger et al. [5] reported a positive association between the self-rated propensity to experience meaningful coincidences and self-rated creative ideation behavior (see also [20]). In accordance with this, Diana et al. [21] found an association between the production of original ideas and the detection of meanings in pictures of natural landscapes. Rominger et al. [22] found that participants with a higher creative potential perceived a higher number of patterns in randomly arranged stimuli in a figural association task. Furthermore, Russo-Netzer and Icekson [23] reported a link between openness and the experience of meaningful coincidences (see also [20]). To sum up, this pattern of findings adds to the assumption that the experience of meaningful coincidences shares variance with several relevant measures of creativity such as creative ideation behavior, creative potential, as well as openness as *the* personality trait linked with creativity [24,25].

However, there is a clear gap in literature and no study to date investigated if people who experience more meaningful coincidences are more often engaged in real-life creativity such as creative activities and creative achievements [11]. This lack of knowledge makes it necessary to conduct this three-study research—assessing a broad range of creativity indices (i.e., creative potential, openness) along with real-life creativity (i.e., creative activities and achievements). This multi-study approach served two main aims. First, we investigated if the experience of meaningful coincidences is robustly associated with real-life creativity (as well as creative potential and openness). Second, we evaluated if PA (and NA) could serve a potential reason for the link between real-life creativity [26] and meaningful coincidences [23]. To achieve the second goal and to study potential mechanisms, we went step-by-step (i.e., from study to study) from a trait perspective (i.e., between-person) to a state perspective (i.e., within-person). The state-of-the-art assessment of more ecologically valid and dynamic data via internet daily diary allows to investigate if affective states as well as affective traits (i.e., PA and NA) might mediate the link between the perception of meaningful coincidences and creative activities (as well as achievements).

## Study 1

Therefore, and as the first step, study 1 investigated if the experience of meaningful coincidences is associated with real-life creativity such as creative activities and creative achievements in different domains like literature, sports, and music [11]. Additionally, we assessed participants’ creative potential, openness, and creative ideation behavior in an online survey to explore creativity relevant associations with the propensity to experience meaningful coincidences.

### Methods

#### Participants

In total 69 participants (45 women) took part in the first study. The mean age was 23.78 years (*SD* = 5.06, min = 18, max = 54). According to self-report, all participants were free of cardiovascular, neurological, or mental disorders as well as psychotropic or cardiovascular medication. Participants were recruited via email and social media. The study was approved by the ethics review board (GZ. 39/100/63 ex 2020/21) and did not take place in a specific context. All participants gave written informed consent to participate in the study by clicking an agree to participate button, which is part of a larger project (see e.g., [27]). The recruitment period of this study was from 01 August 2021 to 31 December 2021. We used Limesurvey for this (Limesurvey GmbH. / LimeSurvey: An Open Source survey tool /LimeSurvey GmbH, Hamburg, Germany. http://www.limesurvey.org).

#### The propensity to experience meaningful coincidences

On the German version of the Coincidence Questionnaire, participants rated how frequently they have experienced several categories of "meaningful" coincidences [2,3,5,28]. The 7 items (e.g., perception of something distant in time such as having a dream that comes true) are rated from "never" to "very often" (5-point Likert scale). The propensity to perceive meaningful coincidences is the sum of all items (*M* = 17.91, *SD* = 4.62, min = 8, max = 28, α = .76).

#### Creative potential

Participants completed the abstract picture fragments of the Test for Creative Thinking–Drawing Production online (TCT-DP; [29]). The time limit was 15 min and was monitored during the online meeting. The generated drawings were then sent to the experimenter. Two independent and trained raters (one man and one woman) scored the TCT-DP in accordance with the test manual (e.g., unconventionality, inclusion of new elements, graphic combinations, etc.). The mean score of both raters was used as index of creative potential (*M* = 1.56, *SD* = 0.54) with a high interrater reliability (*r* = .97).

#### Creative personality (RIBS)

Creative ideation behavior was assessed by a German version of Runco’s Ideational Behavior Scale (RIBS; [30]; see e.g., [11]), which includes 17 statements such as “I come up with an idea or solution other people have never thought of”. Participants responded to the items on a scale ranging from 1 (never) to 5 (very often; *M* = 64.59, *SD* = 14.80; α = .92).

#### Creative activities and creative achievements

We assessed creative activities (CAct) and creative achievements (CAch) with the Inventory of Creative Activities and Achievements (ICAA; 11). The questionnaire asks for 8 different domains of creative activities and achievements (i.e., literature, music, arts and crafts, cooking, sports, visual arts, performing arts, and science and engineering). The creative activities and creative achievements sum scores showed good internal consistency of α = .81 and α = .70 respectively.

#### Openness

We assessed participants’ openness via the NEO-FFI ([31], German translation; [32]). Openness was consistently associated with creative ideation performance and the potential for open problem solving [25,33], as well as real-life creative activities and creative achievements [11]. The internal consistency of openness in the present study was α = .75 (*M* = 34.48, *SD* = 6.51).

#### Statistical analysis

We calculated Pearson correlations to analyze the association between the experience of meaningful coincidences and measures of creativity. To follow-up significant associations between meaningful coincidences and creative activities as well as creative achievements, we calculated Pearson correlations with each sub-score of the ICAA. We calculated all statistical analyses via SPSS (vers. 29; IBM SPSS Statistics, Armonk, NY, USA). The level of significance was *p* < .05 (two-tailed).

### Results

As illustrated in Table 1, the experience of meaningful coincidences was significantly associated with creative activities (*r* = .35, *p* = .003) but failed to reach significance for creative achievements (*r* = .17, *p* = .174). The experience of meaningful coincidences was not associated with openness, creative ideation behavior, and participants’ creative potential (see Table 1). As illustrated in Table 1, creative ideation behavior was significantly associated with creative activities and achievements, which just slightly failed to reach significance for the TCT-DP. Openness was associated with creative activities, creative achievements, and self-rated creative ideation. Creative activities and creative achievements were intercorrelated. This pattern of findings indicates convergent validity of assessment. Age was negatively associated with creative activities and showed a trend for a negative correlation with the experience of meaningful coincidences. When controlling for age, the association between meaningful coincidences and creative activities remained virtually unchanged (*rs* = .310, *p* = .010). Men showed a higher creative potential than women.

**Table 1:** Pearson correlations among variables for study 1.

	Coincidences	CAct	CAch	Openness	Creative potential	RIBS
CAct	**.352** (.003)					
CAch	.165 (.174)	**.623** (< .001)				
Openness	.164 (.178)	.**451** (< .001)	**.409** (< .001)			
Creative potential	-.034 (.782)	.004 (.976)	.225 (.063)	.111 (.363)		
RIBS	.201 (.097)	**.451** (< .001)	**.420** (< .001)	**.408** (< .001)	.120 (327)	
Age	-.235 (.051)	**-.262** (.029)	-.142 (.245)	-.114 (.352)	-.124 (.309)	-.132 (.281)
Gender	.151 (.212)	.207 (.087)	.119 (.329)	.016 (.894)	**-.241 (.046)**	-.211 (.082)

Note. *p* values in parentheses. Creative achievements = Cach, Creative activities = CAct, Test for Creative Thinking–Drawing Production online = TCT-DP, Runco’s Ideational Behavior Scale = RIBS.

Follow-up analysis indicated that the experience of meaningful coincidences was significantly associated with all sub-scores of creative activities, except music and science and engineering (for details see S1 Table). For creative achievements, the correlation coefficients ranged between -.096 and .210 and did not reach significance for any subscale (for details see S1 Table).

### Discussion study 1

In this study, we investigated if the experience of meaningful coincidences is associated with more real-life creativity, which goes beyond studies investigating the association with openness and self-rated creative ideation behavior [5]. In line with the main aim of the study, we found an association between the propensity to experience meaningful coincidences and creative activities such as creating an original talk, writing a blog entry, or making up a rhyme. Creative achievements (as the sum score of 8 different categories) were positively but not significantly associated with the experience of meaningful coincidences (cooking and performing arts showed the highest correlation-coefficients). Nevertheless, for the very first time, this pattern of findings indicates that people who experience more meaningful coincidences are more likely to engage in real-life creative activities. This finding was *not* driven by gender and age and fits the association between positive schizotypy and the engagement in creative behaviors [34].

This novel finding is important since previous work strongly focused on peoples’ creative potential [5,21]. The present study adds a more real-life creativity finding to literature. In line with previous work, the association with self-rated creative behavior and openness showed the expected effect sizes (although not significant). However, we found virtually *no* association between people’s creative potential assessed via the TCT-DP and the propensity to experience meaningful coincidences. This might indicate that the experience of meaningful coincidences is more strongly related to self-rated creative activities but may not be that strongly associated with performance measures of creativity and people’s creative potential. This seems to be in some contrast to the study of Diana et al. [21]. However, it is important to hold in mind that Diana et al. reported an association between two performance measures (i.e., shared methods variance). In their approach, the number of detected meanings in pictures of natural landscapes might capture a potential [21] but not the propensity to perceive meaning (in randomness). In the present study, however, we targeted to assess the *propensity* of meaningful coincidences and found associations with creative activities (and creative achievements).

Of note, creativity indices showed the expected pattern of findings and therefore indicated convergent validity of the assessment. People’s creative potential was related with creative achievements at a trend (see e.g., [35]). Furthermore, creative ideation behavior was associated with other relevant parameters of creativity such as openness, creative activities, as well as creative achievements. In line with literature creative activities were associated with age [11]. Since, the association between meaningful coincidences and real-life creativity constitutes a novel finding, we were interested if this finding could (conceptually) replicate in an independent study 2.

## Study 2

In study 1, we retrospectively assessed the frequency of meaningful coincidences via an established questionnaire and came up with an interesting finding, indicating an association between meaningful coincidences and real-life creativity. However, in study 1, participants rated their frequency of experiencing meaningful coincidences on a scale from “never” to “very often” throughout their entire life. Such a procedure is not a direct assessment of the phenomenon of interest, as people might strongly rely on subjective representations of the frequency of experiences as well as on their beliefs [36,37]. Therefore, this retrospective method might dilute study results and the shared methods variance between questionnaires (e.g., coincidence questionnaire and ICAA) might further lead to an overestimation of effect size of the observed association between the variables of interest.

In order to assess the frequency with which people experience meaningful coincidences when they actually take place and to further increase our trust in the results of study 1, we used an internet daily dairy method in study 2 [37]. The first aim of study 2 was to replicate the association between the propensity to experience meaningful coincidences and real-life creativity. We monitored people’s experience of meaningful coincidences throughout seven days of a week (at most). This procedure allows to assess the dynamic of experiencing meaningful coincidences in more real-time (on a day-to-day basis) and to differentiate between intraindividual and interindividual (i.e., within-person and between-person) differences [37,38]. By applying multilevel models to these micro-longitudinal data, we can differentiate between-person and within-person effects and study cross-sectional and dynamic effects of the experience of meaningful coincidences at the very same time. This is novel in literature, which to date only focused on the interindividual perspective of meaningful coincidences and synchronicity [2,3,23,39–41]. Therefore, the second aim of study 2 is to investigate the fluctuations in the experience of meaning in everyday life for the very first time. In addition to meaningful coincidences, we further assessed participants’ positive and negative affective states throughout the assessment period of a week. This allows to investigate if peoples’ daily affect covaries with the frequency of meaningful coincidences.

First, we can assume that positive affect (PA) constitutes a state of a more flexible and broadened thinking style [42,43], which may be ideal for experiencing meaning in random arrangements. Additionally, the sudden perception of meaningful coincidences might enhance peoples’ PA [23]. Both directions of the effect should go along with a positive association between PA and meaningful coincidences. Second, the perception of meaningful coincidences might serve as a coping strategy. This means that people, who perceive more negative affect (NA) might also experience more meaningful coincidences in order to cope with their negative feelings. In other words, NA affect might increase the “need” to experience meaning in meaningless noise to cope with (random) situations [23]. This means that detecting coincidences might help to find coherence and purpose in life, which might arise from a desire for control ambiguity [28]. This is in line with studies showing associations between the search for meaning and unusual perceptions [44]. Additionally, PA and NA are linked to creativity [26], however, via different pathways [43]. Therefore, we can assume that PA and NA might potentially connect meaningful coincidences with real-life creativity and vice versa. With other words, the affect (PA and NA) might mediate the association between meaningful coincidences and creativity.

To broaden the assessment of creativity, in addition to real-life creative achievements and activities, we again assessed peoples’ openness and their creative potential [45]. Based on the findings of study 1, we assumed a positive association between meaningful coincidences and real-life creativity, although we assessed meaningful coincidences from day to day–which allows the assessment of the trait level of coincidences as well as the dynamic of these experiences.

### Methods

#### Participants

In total, 98 participants took part in the internet daily diary study. Only participants who delivered valid answers for at least three days were included in the final study sample. This resulted in a final sample of 93 participants (74 women) with a mean age of 29.17 years (*SD* = 15.07). All participants gave written informed consent before participating in the online survey by clicking an agree to participate button. The local ethics committee proved this study (GZ. 39/82/63 ex 2014/15), did not take place in a specific context, and the recruitment period of this study was from 1 to 31 May 2022.

#### Daily diary method

Participants received a daily email with a link to the online survey that included 8 questions assessing the experience of meaningful coincidences and the 20 items of the PANAS to assess their daily affect. We used Limesurvey for this (Limesurvey GmbH. / LimeSurvey: An Open Source survey tool /LimeSurvey GmbH, Hamburg, Germany. http://www.limesurvey.org).

#### Experience of meaningful coincidences

Participants were asked how often they had perceived meaningful coincidences during the day by means of 8 items. They had to report the number of meaningful experiences they have experienced throughout the day in 7 different categories (taken from the coincidence questionnaire). Furthermore, we assessed other types of coincidences with one additional item (i.e., How many coincidences of other domains did you experience today?). For all calculations, we transformed the number of coincidences per day into a 5-point ordinal scale from 0 to 4 (i.e., 4 and more experiences per day). The mean number of coincidences per day was *M* = 0.25 (*SD* = 0.32) ranging from 0 to 2. The mean number of coincidences at the between-person level was *M* = 0.25 (*SD* = 0.26) ranging from 0 to 1.41. By applying generalizability theory analysis (GTA; [46]; for an application see e.g., [47]), we indicated excellent reliability for the assessment of the number of coincidences at the between-person level (R_kR_ = .92) and acceptable reliability at the within-person level with R_C_ = .39 [48].

#### Affect

Furthermore, each day we asked participants to answer all 20 items of the German version of the Positive and Negative affect Schedule (PANAS; [49]). The mean PA per day was *M* = 2.79 (*SD* = 0.80; NA: *M* = 1.54, *SD* = 0.56) ranging from 1 to 5 (NA: 1 to 4.1). The mean PA at the between-person level was *M* = 2.78 (*SD* = 0.55; NA: *M* = 1.55, *SD* = 0.40) ranging from 1.35 to 4.50 (NA: 1 to 2.62). The reliability of the PA was good for between-person (R_kR_ = .84) and for within-person (R_C_ = .90). Similarly, NA was assessed reliable with R_kR_ = .85 for between-person and R_C_ = .82 for within-person variation.

### Online assessment of trait variables

#### Creative activities and creative achievements

Creative activities (CAct) and creative achievements (CAch) were assessed by means of the Inventory of Creative Activities and Achievements (ICAA; [11]). This questionnaire asks for eight different domains of creative activities and achievements (i.e., literature, music, arts & crafts, cooking, sports, visual arts, performing arts, and science and engineering). The sum score of creative activities showed an internal consistency of α = .79 and creative achievements score showed a α of .73.

#### Coincidence questionnaire

We used the same coincidence questionnaire as in study 1. The Cronbach alpha was α = .78. This questionnaire served as validity measure for the daily diary assessment of meaningful coincidences with *M* = 17.45 (*SD* = 4.91).

#### Creative potential

We used the divergent association task (DAT; [45]) to assess peoples’ creative potential. Participants were instructed to produce 10 unrelated words within 4 minutes. The unrelatedness served as an indicator of people’s creative potential. To calculate the distance between the words, we translated the German words into English. For the translated words we calculated the distance measures by means of the provided online application [45]. The mean distance score was 80.37 (*SD* = 5.10).

#### Openness

Openness was assessed via the German version of the Big Five Inventory 2 (BFI-2; [50]), which assesses three sub-facets of openness (i.e., intellectual curiosity, aesthetic sensitivity, creative imagination). Cronbach alpha of the total score was α = .84.

#### Procedure

Before the daily diary assessment all participants filled in the online questionnaires and worked on an online version of the DAT (LimeSurvey GmbH, Hamburg, Germany). Then participants registered for a daily dairy assessment, where they received an email per day with a link to the daily survey. Participants should answer the daily diary each day between 6 pm and 12 pm.

#### Statistical analyses

First, to evaluate the proportion of variance associated with between-person and within-person variance for PA, NA, and meaningful coincidences respectively, we applied generalizability theory analyses (GTA; [46]) by the use of the software psych (Version 2.3.3; [51]) running in R (Version 3.4.2; [52]). Following Netzlek [48], we differentiated between Level 1 (items), Level 2 (days), and Level 3 (person). GTA is especially suited to assess reliability of daily-diary data, allowing the partitioning of between-person, within-person, and error variance by decomposing the observed variance associated with person, item, day, and their respective interactions. Second, we calculated Pearson correlations to investigate if the number of experienced meaningful coincidences assessed in everyday life was significantly associated with real-life creativity (and the other measures of creativity, i.e., creative potential and openness). Third, to investigate if the association between meaningful coincidences and real-life creativity was mediated by PA (and NA), we calculated mediation analyses with the lavaan package (vers. 0.6–14). Third, to evaluate the association between meaningful coincidences and affect from a trait as well as a state perspective, we calculated a multilevel model with PA and NA as fixed effects predicting the number of meaningful coincidences per day. In this robust multilevel model, we used participants as random factor and level 2 (grand mean centered) and level 1 (group mean centered) affect (i.e. PA and NA) as fixed effects. We applied robust linear mixed effects modeling (package robustlmm; [53]) using R [52] to account for potentially biasing outliers. The level of significance was fixed at *p* < .05 (two-tailed).

### Results

The experience of meaningful coincidences at the aggregated between-person level was significantly associated with the score on the coincidence questionnaire (*r* = .38, *p* < .001). This indicates validity of the frequency of meaningful coincidences assessed via the daily diary method. 93% of participants experienced at least one meaningful coincidence throughout the assessment. As illustrated in Table 2, 14% of variance of meaningful coincidences was between-person and 5% of variance was due to day-to-day variance (i.e., interaction between person and time; see Table 2). The proportion of between-person variance was comparable for PA and NA.

**Table 2:** Proportion of variance at the between-person and the within-person level for all variables assessed via internet daily diary.

	Coincidences		PA		NA	
Variance component						
$\sigma_{Total}^{2}$	0.35		1.19		0.78	
$\sigma_{P}^{2}$	0.05	14%	0.23	20%	0.12	15%
$\sigma_{T}^{2}$	0.00	0%	0.00	0%	0.00	0%
$\sigma_{I}^{2}$	0.01	3%	0.08	7%	0.07	8%
$\sigma_{P*T}^{2}$	0.02	5%	0.34	29%	.015	19%
$\sigma_{P*I}^{2}$	0.08	8%	0.13	11%	0.12	15%
$\sigma_{T*I}^{2}$	0.00	0%	0.00	0%	0.00	0%
$\sigma_{Residuals}^{2}$	0.20	57%	0.40	33%	0.33	42%
GT reliability estimates						
*R*_*KR*_	.92		.84		.85	
*R*_*C*_	.39		.90		.82	

*Note*. *σ*^2^ = variance component, P = person, T = time, I = item; *R*_*KR*_ = between-person reliability; *R*_*C*_ = within-person reliability.

#### Meaningful coincidences and creativity

**Fig 1:**
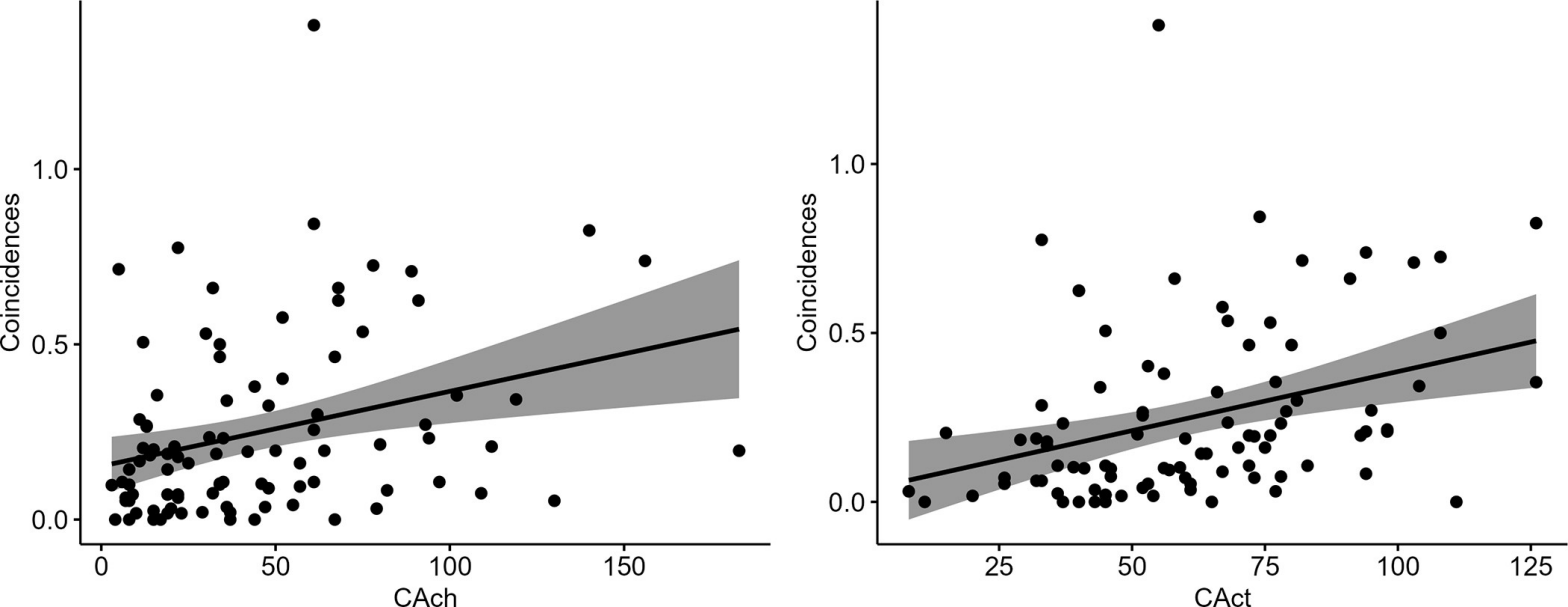
Pearson correlations between the frequency of meaningful coincidences and the two real-life creativity measures.

Creative achievements (*r* = .31, *p* = .003) and creative activities (*r* = .35, *p* < .001) were significantly associated with the number of coincidences experienced during the seven days of the daily diary assessment (see Fig 1).

Follow-up analyses indicated that for creative activities this association was driven by literature, music, arts and crafts, as well as sports (see S1 Table). For creative achievements, the follow-up analyses indicated that the association was driven by music, cooking, sports, and performing arts (see S1 Table).

As illustrated in Table 3, Openness was not associated with the experience of coincidences (*r* = .12, *p* = .233). Age was negatively associated with NA and creative achievements. Therefore, the association between creative achievements/activities and coincidences are virtually unchanged when controlling for age.

**Table 3:** Pearson correlations among variables for study 2.

* *	*PA*	*NA*	*CAct*	*CAch*	*Openness*	*DAT*	*Age*	*Gender*
*Meaningful coincidences*	0.253 *(*.*014)*	0.204 *(*.*050)*	0.345 *(*.*001)*	0.306 *(*.*003)*	0.125 *(*.*233)*	0.161 *(*.*126)*	-0.114 *(*.*276)*	-0.026 *(*.*808)*
*PA*		-0.229 *(*.*027)*	0.028 *(*.*790)*	0.106 *(*.*313)*	-0.016 *(*.*878)*	0.166 *(*.*113)*	0.167 *(*.*109)*	0.069 *(*.*511)*
*NA*	-0.229 *(*.*027)*		0.288 *(*.*005)*	0.178 *(*.*087)*	0.034 *(*.*748)*	0.035 *(*.*743)*	-0.244 *(*.*018)*	-0.165 *(*.*114)*
*CAct*	0.028 *(*.*790)*	0.288 *(*.*005)*		0.591 *(<* .*001)*	0.366 *(<* .*001)*	0.093 *(*.*377)*	-0.152 *(*.*145)*	-0.118 *(*.*260)*
*CAch*	0.106 *(*.*313)*	0.178 *(*.*087)*	0.591 *(<* .*001)*		0.253 *(*.*015)*	0.225 *(*.*031)*	-0.268 *(*.*009)*	-0.075 *(*.*474)*
*Openness*	-0.016 *(*.*878)*	0.034 *(*.*748)*	0.366 *(<* .*001)*	0.253 *(*.*015)*		0.168 *(*.*110)*	-0.015 *(*.*886)*	-0.063 *(*.*549)*
*DAT*	0.166 *(*.*113)*	0.035 *(*.*743)*	0.093 *(*.*377)*	0.225 *(*.*031)*	0.168 *(*.*110)*		-0.159 *(*.*130)*	-0.073 *(*.*492)*

Note. Creative achievements = CAch, Creative activities = CAct, divergent association task = DAT, positive affect = PA, negative affect = NA.

Furthermore, PA and NA were positively related with the number of meaningful coincidences. Only NA was significantly related to creative activities. The creative potential was associated with creative achievements indicating some validity.

#### Mediating effects of affect on the association between creativity and meaningful coincidences

The mediation analysis indicated no significant indirect path of coincidences via PA to creative activities (Beta = -1.58, *p* = .540). The total effect (direct plus indirect) was significant (Beta = 34.00, *p* < .001). We observed a similar pattern for NA, with no significant indirect path (Beta = 4.66, *p* = .127; total effect: Beta = 34.00, *p* < .001). This pattern of findings argues for independent associations of coincidences and PA with creative activities.

#### A dynamic perspective on meaningful coincidences

The robust multilevel model indicated that more meaningful coincidences were perceived on days on which participants showed higher PA (Level 1; Beta = 0.08, *p* < .001). People with a higher trait PA (at Level 2) also perceived more coincidences in general (Beta = 0.11, *p* = .004). Similarly, NA was significantly associated with meaningful coincidences at Level 2 (Beta = 0.15, *p* = .004) and at Level 1 (Beta = 0.04, *p* = .029).

### Discussion study 2

Study 2 replicated the association between the experience of meaningful coincidences and real-life creativity such as creative activities and creative achievements. This finding fits the results of Baas et al. [34], who reported an association between positive schizotypy and creative achievements. Furthermore, the experience of meaningful coincidences was again, unrelated to the measure of creative potential. For the very first time this study applied an internet daily diary method to assess the frequency of meaningful coincidences and found that people perceive a considerable number of meaningful coincidences within a week on a day-to-day basis. Furthermore, the highest proportion of the number of experienced coincidences variance was between-person (14%), which underlines the assumption that meaningful coincidences represent a trait, which we can reliably assess in everyday life by means of daily diary [2,3]. The within-person variance was assessed at the lower boundary of reliability [48]. However, this proportion of variance (i.e., 5%) was meaningfully associated with fluctuations of people’s affect, arguing for validity of assessment. Exactly during days on which participants experienced more PA and NA, they also experienced more meaning in random arrangements. This is partly in line with a study of Conner and Silva [54], who conducted a two-week internet daily diary study and showed that PA states were associated with creative behavior and creative activity [55–57].

The association between PA states and the number of experienced meaningful coincidences underlines the validity of the assessment and offers a novel state perspective on the link between PA and the perception of meaning in everyday life [23]. Not only peoples’ affect fluctuates from day-to-day, but their experience of meaningful coincidences seems to do so as well, and both seem to be related. Furthermore, the traits of PA and NA were associated with the number of meaningful coincidences througout the week. This is in accordance with cross-sectional research indicating a link between positive feelings, well-being, and life-satisfaction with the experience of more meaningful coincidences [23]. The correlation with NA argues for the assumption that the experience of meaningful coincidences may serve as a coping mechanism [28]. Similar to this finding, Russo-Netzer and Icekson [23] reported a positive link between coincidences and depressive symptoms (in a non-clinical sample). They suggested that the experience of meaningful coincidences (and synchronicity) may serve as one pathway to life satisfaction. The relationship with PA fits this assumption well.

The observed pattern of findings is convincing since PA and NA were entered simultaneously into our model. This approach rules out a simpler interpretation that the positive association between meaningful coincidences and PA and NA was because of a conformation bias or the tendency of perceivers to say “yes” due to a lower threshold for giving positive answers [18,58]. This would have affected all daily diary ratings to a similar extend, which would have resulted in non-significant findings.

Importantly, neither PA nor NA seems to serve as a mechanism explaining why people who perceive more meaningful coincidences also showed more real-life creativity. Study 2 investigated this association on the between-person level. Consequently, we conducted a study 3 to provide a more detailed look into the within-person dynamics of meaningful coincidences, affect, and creative activities.

## Study 3

In study 3, our first goal was to replicate the association between creative activities (and achievements) with the experience of meaningful coincidences. Based on the study findings outlined above, we pre-registered study 3 [59]. The second goal was to extend this finding and provide a conceptual replication by additionally using a different measure of the daily experience of synchronicity [23] and by assessing the creative activities for each day–again increasing ecological validity and reducing recall biases in the data.

As the third aim, we studied if creative activities and the experience of meaningful coincidences would show a dynamic relationship from day to day. Based on this relationship we targeted to investigate affect (PA and NA) as a potential mechanism why people show more creative activities when experiencing more meaningful coincidences (and vice versa; on a day-to-day basis). PA might serve a valuable mechanism because it goes along with more creative activities (e.g., [57]) and meaningful coincidences (see study 2). Furthermore, also NA shows associations with meaningful coincidences (see study 2) and is (theoretically) linked to creativity as well [43].

To sum up, study 3 assessed real-life creativity, creative potential, and openness cross-sectionally by means of questionnaires, and additionally measured the dynamic of meaningful coincidences, affect, and creative activities by means of an internet daily diary. We preregistered the cross-sectional association between meaningful coincidences and real-life creativity [59] and targeted a conceptual replication as well. Furthermore, we investigated if affect (PA, NA) might mediate the association between meaningful coincidences and creative activities at the within and the between-person level.

### Methods

#### Participants

All data and scripts of Study 3 are available online [59]. We planned to collect a sample of at least 80 participants for up to seven days, which should be sufficient to find a significant Pearson correlation of medium size (*r* = .30) with a power of .80 and an alpha of .05. We only analyzed participants who answered at least 3 daily diaries. The final sample comprised 80 participants with a mean age of 29.66 years (*SD* = 12.82). In total, 35 men, 44 women, and one diverse person participated in the study, which did not take place in a specific context. The local ethics committee approved the study (GZ. 39/82/63 ex 2014/15), the recruitment period of this study was from 01 May to 30 June 2023, and all participants gave written informed consent by clicking an agree to participate button.

#### Procedure of the daily diary assessment

Participants received a daily email with a link to the online survey including 8 questions on the experience of meaningful coincidences, 20 items of the PANAS, 8 questions asking for creative activities, and further 9 items assessing synchronicity (a concept similar to meaningful coincidences). The study started on a Monday, where we also applied the cross-sectional questionnaires, and ended on a Sunday. Participants had to answer the daily diary each day between 6 pm and 12 pm. We used LimeSurvey to apply the questionnaires (LimeSurvey GmbH, Hamburg, Germany).

#### Coincidences questionnaire

We used the 8 items from study 2 [3]. Participants answered how often they perceived specific meaningful coincidences during the day. In accordance with study 2, we transformed the number of coincidences per day into a 5-point ordinal scale from 0 to 4. The mean number of perceived meaningful coincidences per day on the between-person level was 0.22 (*SD* = 0.28) ranging from 0 to 1.5. The mean number of coincidences per day was 0.20 (*SD* = 0.32) ranging from 0 to 1.88. Again between-person reliability was excellent (R_kR_ = .94), and within-person level was acceptable R_C_ = .42.

#### Synchronicity awareness

To assess synchronicity (i.e., meaningful coincidences), we used the German translation of the Synchronicity Awareness scale [23]. Similar to the coincidence questionnaire [3], the SA scale referred to awareness of the occurrence of synchronicity events and involves 9 items. We used this newly developed assessment approach, see if results can be replicated between the two coincidence questionnaires. We used a 5-point Likert Scale from 0 to 4 (i.e., no synchronicity experience, 4 and more experiences per day). The mean number of synchronicity events per day on the between-person level was 0.34 (*SD* = 0.28) ranging from 0 to 1.37. The mean score per day was 0.32 (*SD* = 0.36) ranging from 0 to 3.11 The between-person (R_kR_ = .86) and within-person reliability (R_C_ = .62) were good.

#### Creative activities

We asked participants to rate if they had performed creative activities on 8 different domains taken from the ICAA [11]. We calculated the sum score of creative activities per day. At the day level the mean score was 1.16 (*SD* = 1.21) ranging from 0 to 7. At the between-person level the mean score was 1.24 (*SD* = 0.97) ranging from 0 to 4.25. People reported at least one creative activity on 61.20% of the days. Creative activities were assessed reliable with R_kR_ = .90 for between-person. The within-person reliability was low with R_C_ = .13.

#### Positive and negative affect

Again, we asked participants to answer all 20 items of the German version of the Positive and Negative affect Schedule (PANAS; [49]). The mean PA per day was *M* = 2.91 (*SD* = 0.79; NA: *M* = 1.40, *SD* = 0.47) ranging from 1 to 5 (NA: 1 to 4.2). The mean PA at the between-person level was *M* = 2.92 (*SD* = 0.57; NA: *M* = 1.42, *SD* = 0.35) ranging from 1.69 to 4.55 (NA: 1 to 2.55). The reliability of the positive affect was good for between-person (R_kR_ = .84) and for within-person (R_C_ = .89). Similarly, negative affect was assessed reliable with R_kR_ = .85 for between-person and R_C_ = .78 for within-person variation.

### Online assessment of trait variables

#### Creative activities and creative achievements

Creative activities (CAct) and creative achievements (CAch) were assessed by means of ICAA [11]. The sum score of creative activities showed an internal consistency of α = .75 (creative achievements: α = .71).

#### Creative potential

We used the alternate uses task (AUT; [60]) to assess peoples creative potential. Participants were instructed to produce as many creative uses as possible for the objects brick and paperclip within a 3-minute time limit for each. The Spearman-Brown corrected reliability of both items was .86. The mean fluency score was 6.24 (*SD* = 3.03). To calculate the originality, we translated the ideas from German into English via google translate and consequently checked them. For the translated ideas, we calculated distance scores by means of the online application SemDis. We used multiplicative models for semantic distance computations [61] and calculated the maximum score of the semantic distance factor per item as a measure of originality. The mean score showed a Spearman-Brown corrected reliability of .56. The originality score was 0.08 (*SD* = 0.04).

#### Openness to experiences

Openness to experiences was assessed via the German version of the BFAS-G with 10 items [62]. The items were answered on a seven-point Likert scale. Cronbach alpha was α = .79.

#### Statistical analyses

First, and in accordance with study 2, we evaluated the proportion of variance associated with between-person and within-person variance for PA, NA, meaningful coincidences, synchronicity, and creative activities by applying the GTA [46] by the use of the software psych (Version 2.3.3; [51]) running in R (Version 3.4.2; [52]). Second, to replicate the findings of study 1 and 2 we calculated single-order Pearson correlations between meaningful coincidences and real-life creativity (and the other aspects of creativity). Third, to investigate if PA and NA (from a state and trait perspective) were associated with meaningful coincidences, we calculated multilevel models predicting coincidences. Fourth, to investigate if meaningful coincidences are associated with the fluctuation of creative activities from day to day, we calculated multilevel models predicting creative activities by means of coincidences. Fifth, we calculated multilevel models predicting creative activities by means of affect. We used robust linear mixed effects models (package robustlmm; [53]) using R [52] for all multi-level models. Finally, to investigate potential mediating effects of PA and NA for the association between meaningful coincidences and creative activities, we calculated mediation analyses taking the between-person (2-2-2) and within-person (1-1-1) level into account. We used the lavaan package (vers. 0.6–14). As a conceptual replication attempt, all analyses were also calculated for synchronicity awareness. The level of significance was fixed at *p* < .05 (two-sided).

### Results

The experience of meaningful coincidences at the aggregated between-person level was significantly associated with the synchronicity awareness score (*r* = .76, *p* < .001). Only 6 participants perceived no single meaningful coincidence. 20% of variance of meaningful coincidences was between-person and 5% were due to day-to-day fluctuations. For synchronicity, the between-person was 10% and the within-person variances was 9%. Here, only 2 participants had no synchronicity experience at all (see Table 4). The proportions of variance for PA and NA were comparable to study 2. Creative activities showed 7% of variance between-person and only 1% within-person.

**Table 4:** Proportion of variance at the between-person and the within-person level for all variables assessed via internet daily diary.

	Coincidences		Synchronicity		PA		NA		CAct_daily	
Variance component										
$\sigma_{Total}^{2}$	0.33		0.49		1.21		0.58		0.13	
$\sigma_{P}^{2}$	0.07	20%	0.05	10%	0.24	20%	0.09	15%	0.01	7%
$\sigma_{T}^{2}$	0.00	0%	0.00	0%	0.00	0%	0.00	0%	0.00	0%
$\sigma_{I}^{2}$	0.01	3%	0.04	9%	0.07	5%	0.04	7%	0.01	7%
$\sigma_{P*T}^{2}$	0.02	5%	0.05	9%	0.34	28%	0.10	18%	0.00	1%
$\sigma_{P*I}^{2}$	0.06	18%	0.09	19%	0.14	11%	0.07	11%	0.03	23%
$\sigma_{T*I}^{2}$	0.00	0%	0.00	0%	0.00	0%	0.00	0%	0.00	0%
$\sigma_{Residuals}^{2}$	0.17	54%	0.26	53%	0.43	36%	0.29	49%	0.08	62%
GT reliability estimates										
*R*_*KR*_		.94		0.86		.84		.85		.90
*R*_*C*_		.42		0.62		.89		.78		.13

*Note*. *σ*^2^ = variance component, P = person, T = time, I = item; *R*_*KR*_ = between-person reliability; *R*_*C*_ = within-person reliability.

#### Meaningful coincidences and real-life creativity

**Fig 2:**
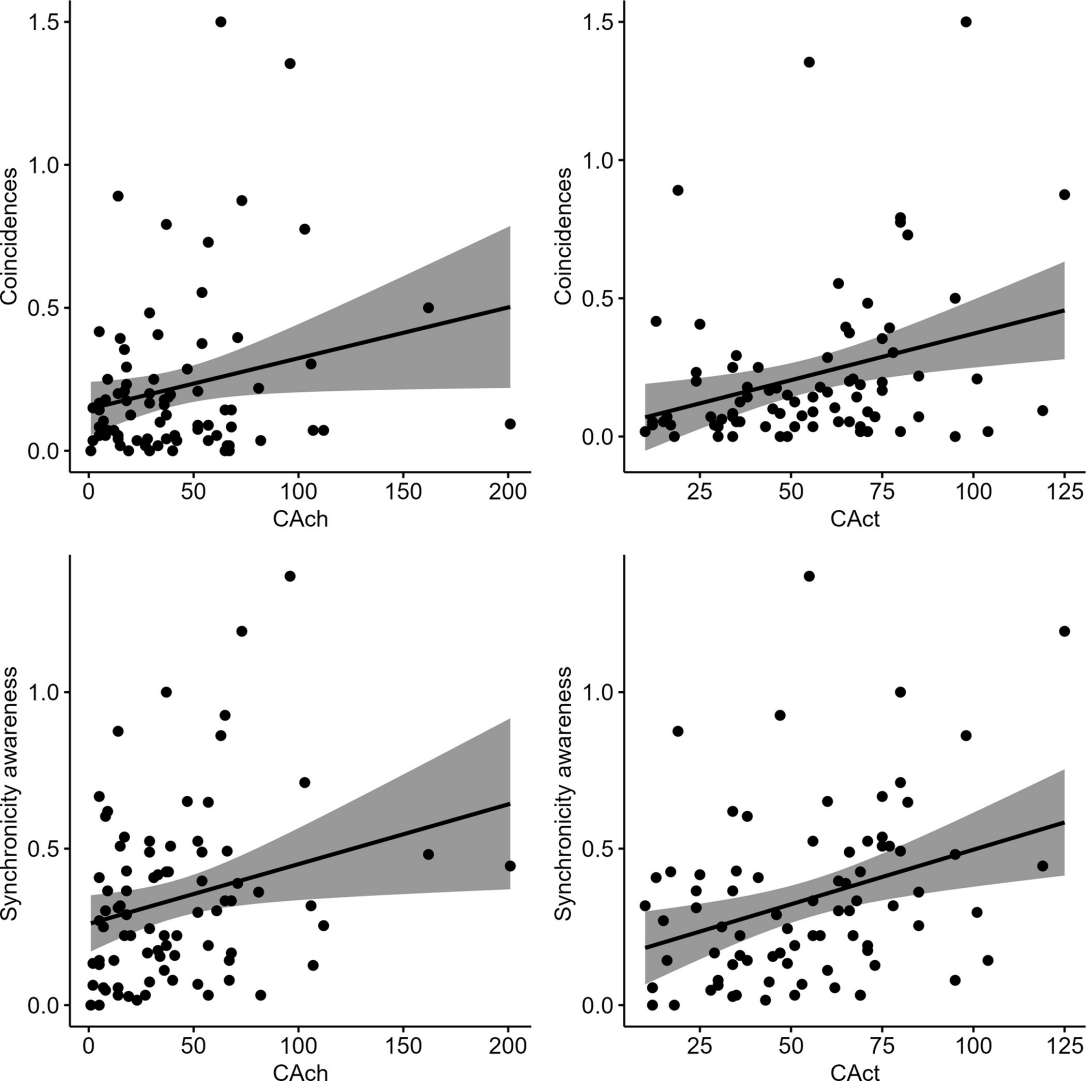
Pearson correlations between the frequency of the two real-life creativity measures and meaningful coincidences top panel and synchronicity awareness at the bottom panel.

Creative achievements (*r* = .23, *p* = .044) and creative activities (*r* = .31, *p* < .001) were significantly associated with the number of coincidences experienced during the seven days of the daily diary assessment. We found a similar pattern for synchronicity awareness (see Fig 2). Follow-up analyses indicated that the significant finding for creative activities was driven by literature, music, sports, visual arts, and performing arts (see S1 Table). For creative achievements, the finding was mainly due to cooking, visual arts, and performing arts (see S1 Table).

As illustrated in Table 5, age was significantly associated with NA and creative achievements, however, not with meaningful coincidences and synchronicity. Therefore, age does not substantially affect the correlation between coincidences/synchronicity and creative activities/achievements. Gender showed no significant association.

**Table 5:** Pearson correlations among variables for study 3.

	*Daily diary*				*Cross-sectional*						
* *	*Synchronicity*	*PA*	*NA*	*CAct_daily*	*CAct*	*CAch*	*Openness*	*AUT-Flu*	*AUT-Ori-Top*	*Age*	*Gender*
*Meaningful coincidences*	0.765 *(<* .*001)*	0.395 *(<* .*001)*	0.270 *(*.*016)*	0.475 *(<* .*001)*	0.307 *(*.*006)*	0.226 *(*.*044)*	0.101 *(*.*374)*	0.067 *(*.*557)*	-0.011 *(*.*921)*	-0.126 *(*.*266)*	-0.135 *(*.*235)*
*Synchronicity*		0.432 *(<* .*001)*	0.220 *(*.*050)*	0.529 *(<* .*001)*	0.328 *(*.*003)*	0.250 *(*.*025)*	0.158 *(*.*161)*	0.000 *(*.*997)*	-0.042 *(*.*711)*	0.070 *(*.*537)*	-0.030 *(*.*792)*
*PA*	0.432 *(<* .*001)*		-0.056 *(*.*624)*	0.298 *(*.*007)*	0.284 *(*.*011)*	0.322 *(*.*004)*	0.148 *(*.*189)*	0.187 *(*.*097)*	0.218 *(*.*052)*	0.005 *(*.*967)*	-0.097 *(*.*396)*
*NA*	0.220 *(*.*050)*	-0.056 *(*.*624)*		0.058 *(*.*609)*	0.276 *(*.*013)*	0.188 *(*.*095)*	0.093 *(*.*411)*	-0.006 *(*.*957)*	0.040 *(*.*724)*	-0.359 *(*.*001)*	-0.074 *(*.*516)*
*CAct_daily*	0.529 *(<* .*001)*	0.298 *(*.*007)*	0.058 *(*.*609)*		0.499 *(<* .*001)*	0.417 *(<* .*001)*	0.322 *(*.*004)*	0.051 *(*.*656)*	0.045 *(*.*689)*	-0.204 *(*.*070)*	0.021 *(*.*854)*
*CAct*	0.328 *(*.*003)*	0.284 *(*.*011)*	0.276 *(*.*013)*	0.499 *(<* .*001)*		0.633 *(<* .*001)*	0.407 *(<* .*001)*	0.280 *(*.*012)*	-0.009 *(*.*937)*	-0.202 *(*.*072)*	-0.013 *(*.*906)*
*CAch*	0.250 *(*.*025)*	0.322 *(*.*004)*	0.188 *(*.*095)*	0.417 *(<* .*001)*	0.633 *(<* .*001)*		0.380 *(*.*001)*	0.341 *(*.*002)*	0.105 *(*.*355)*	-0.256 *(*.*022)*	-0.041 *(*.*721)*
*Openness*	0.158 *(*.*161)*	0.148 *(*.*189)*	0.093 *(*.*411)*	0.322 *(*.*004)*	0.407 *(<* .*001)*	0.380 *(*.*001)*		0.253 *(*.*024)*	0.016 *(*.*887)*	-0.012 *(*.*916)*	0.034 *(*.*766)*
*AUT-Flu*	0.000 *(*.*997)*	0.187 *(*.*097)*	-0.006 *(*.*957)*	0.051 *(*.*656)*	0.280 *(*.*012)*	0.341 *(*.*002)*	0.253 *(*.*024)*		0.347 *(*.*002)*	-0.116 *(*.*306)*	0.040 *(*.*728)*
*AUT-Ori-Top*	-0.042 *(*.*711)*	0.218 *(*.*052)*	0.040 *(*.*724)*	0.045 *(*.*689)*	-0.009 *(*.*937)*	0.105 *(*.*355)*	0.016 *(*.*887)*	0.347 *(*.*002)*		-0.085 *(*.*453)*	-0.062 *(*.*586)*

Note. *p* values in parentheses. Creative achievements = Cach, Creative activities = CAct, alternate uses task = AUT, positive affect = PA, negative affect = NA.

Furthermore, openness was not associated with coincidences. PA and NA were positively associated with the number of meaningful coincidences as well as the synchronicity score. NA affect was significantly related to creative activities. PA affect and openness were associated with creative activities and achievements. The creative potential measured via fluency was associated with creative activities, achievements as well as openness. The semantic distance score was associated with the fluency score and at a trend with PA. This pattern of findings indicates some validity of measures.

#### The association between meaningful coincidences and affect

The robust multilevel model indicated that people with a higher PA as well as higher NA as a trait (at Level 2) also perceived more coincidences in general. PA each day (Level 1) and as a trait (Level 2) also predicted the experience of synchronicity (see Table 6).

**Table 6:** Multilevel model predicting coincidences/synchronicity via affect.

	Coincidences			Synchronicity		
*Predictors*	*Estimates*	*Conf*. *Int (95%)*	*p-value*	*Estimates*	*Conf*. *Int (95%)*	*p-value*
Intercept	-0.30	-0.52 – -0.07	**0.010**	-0.31	-0.62 – 0.01	0.054
PA (trait Level 2)	0.08	0.02 – 0.13	**0.010**	0.16	0.08 – 0.24	**<0.001**
PA (state Level 1)	0.02	-0.01 – 0.04	0.140	0.08	0.05 – 0.11	**<0.001**
NA (trait Level 2)	0.16	0.06 – 0.26	**0.001**	0.09	-0.04 – 0.22	0.182
NA (state Level 1)	-0.02	-0.05 – 0.02	0.336	0.04	-0.01 – 0.09	0.146
**Random Effects**						
σ^2^	0.02			0.03		
τ_00_	0.02 _Participant_			0.04 _Participant_		
ICC	0.55			0.55		
N	80 _Participant_			80 _Participant_		
Observations	482			482		
Marginal R^2^ / Conditional R^2^	0.119 / 0.601			0.138 / 0.615		

#### The association between meaningful coincidences and creative activities

As illustrated in Table 7, the robust multilevel model indicated that meaningful coincidences as a trait (Level 2) predicted creative activities in everyday life. A similar pattern was found for synchronicity awareness, which additionally predicted creative activities from day to day.

**Table 7:** Multilevel model predicting creative activities via coincidences/synchronicity.

	CAct_daily—Coincidences			Cact_daily—Synchronicity		
*Predictors*	*Estimates*	*Conf*. *Int (95%)*	*p-value*	*Estimates*	*Conf*. *Int (95%)*	*p-value*
Intercept	0.79	0.54 – 1.03	**<0.001**	0.51	0.22 – 0.80	**0.001**
Trait (Level 2, person)	1.73	1.03 – 2.43	**<0.001**	1.99	1.31 – 2.67	**<0.001**
State (Level 1, day)	0.19	-0.20 – 0.58	0.334	0.34	0.06 – 0.61	**0.018**
**Random Effects**						
σ^2^	0.57			0.58		
τ_00_	0.66 _Participant_			0.57 _Participant_		
ICC	0.53			0.50		
N	80 _Participant_			80 _Participant_		
Observations	482			482		
Marginal R^2^ / Conditional R^2^	0.148 / 0.603			0.195 / 0.595		

#### The association between creative activities and affect

The robust multilevel model indicated that state PA (Level 1, Beta = 0.20, *p =* .002) and trait PA (Level 2; Beta = 0.48, *p =* .019) predicted creative activities in everyday life, but not NA (Level 1: Beta = -0.06, *p =* .565; Level 2: Beta = 0.27, *p =* .427).

#### The mediating effect of affect on the association between meaningful coincidences/synchronicity and creative activities in everyday life

**Fig 3:**
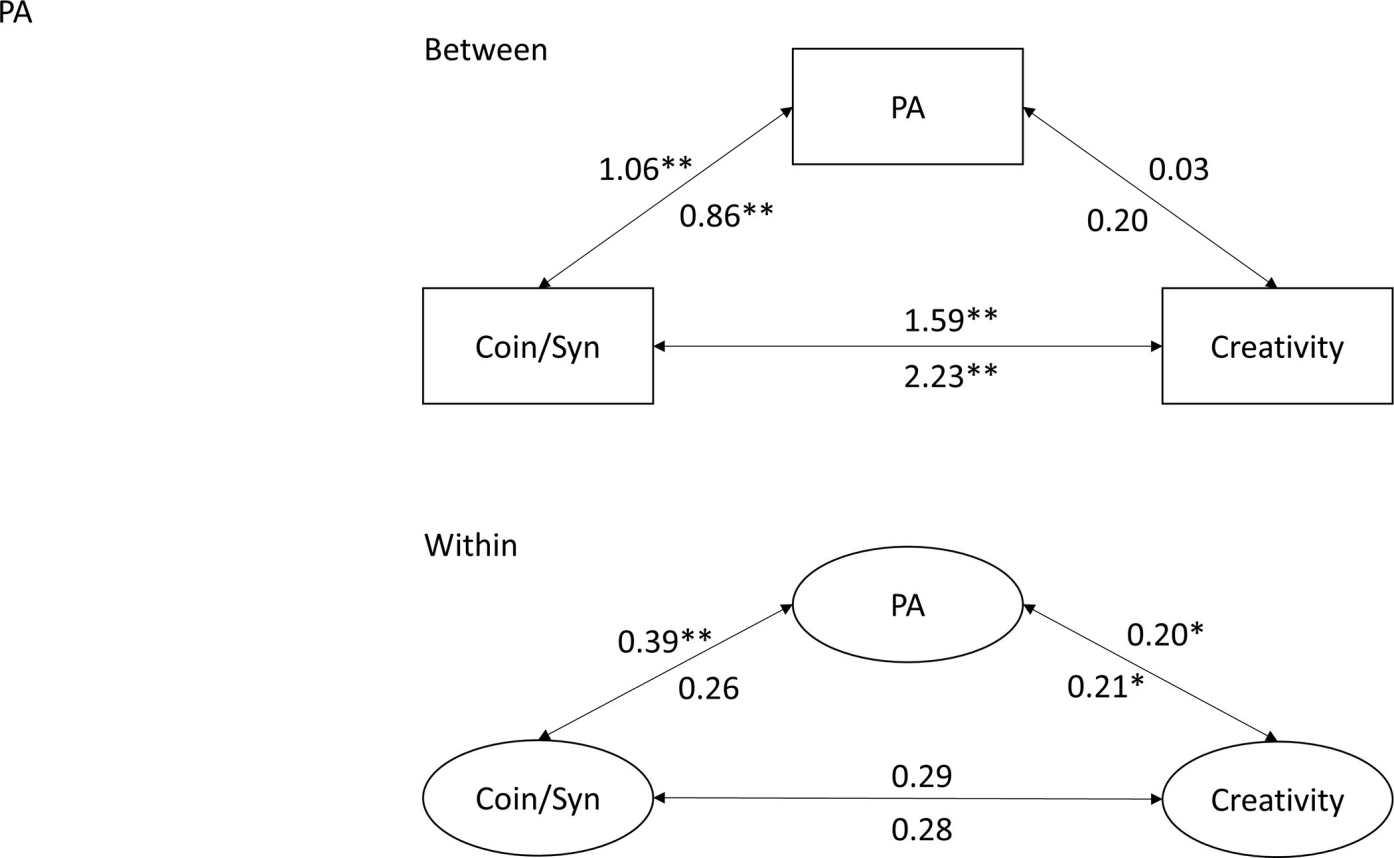
Between and within mediation analyses with Beta of meaningful coincidences in the inner and synchronicity in the outer.

The mediation analysis indicated no significant indirect path of meaningful coincidences via PA to creative activities, neither for the between-person Level 2 (Beta = 0.17, *p* = .423), nor for the within-person Level 1 (Beta = 0.05, *p* = .150). The total effect (direct plus indirect) was significant for between-person (Beta = 1.76, *p* < .001) but not for within-person (Beta = 0.35, *p* = .113; see Fig 3). This pattern of findings argues for an independent association of coincidences and PA with creative activities at both levels of analysis.

For synchronicity, we found a very similar pattern. However, the indirect path of synchronicity via PA to creative activities was significant for within-person (Beta = 0.08, *p* < .027) and the direct effect to creativity was not (Beta = 0.28, *p* = .068). This indicates a within-person mediation effect of the association between synchronicity and creative activities (see Fig 3).

We found no significant mediation effect of NA neither for meaningful coincidences nor for synchronicity.

## Discussion of study 3

As preregistered, study 3 replicated the findings of study 1 and study 2. The propensity to experience meaningful coincidences was again related to real-life creativity such as creative activities and creative achievements. Furthermore, we were also able to conceptually replicate this pattern in a twofold manner. First, by means of an alternative questionnaire developed by Russo-Netzer and Icekson [23] applied in a daily-diary context and second, by assessing creative activities from day to day [57]. Both modifications showed the very same pattern of findings–that there is a link between synchronicity and creative activities at the between-person level.

Well in line with literature, PA at day level predicted the number of creative activities participants were engaged in [54,55,57]. However, creative activities on a day-to-day basis were not that strongly associated with meaningful coincidences. People who perceive more meaning in randomly arranged events are likely more often engaged in creative activities and report more creative achievements at the trait level. This strengthens the interpretation of the experience of meaningful coincidences as a propensity [2,3]. By applying an internet daily diary assessment, we were able to decrease potential influences of memory and belief processes on our findings [37].

However, study 3 did not replicate the finding of study 2, which had suggested that the dynamic of PA and NA from day to day is associated with the experience of meaningful coincidences. However, of note, we found an association of PA state and synchronicity awareness. The mediation effect on the association between synchronicity and creative activities was only found for PA at the within-person level. Therefore, it seems promising to conduct further investigations on why and when (positive) affective states might impact the experience of meaningful coincidences.

### General discussion

In this three-study approach, we repeatedly found that the experience of meaningful coincidences is associated with real-life creativity such as creative activities (three out of three studies) and creative achievements (two out of three studies). This association was firstly found in a cross-sectional online study, then replicated in a consecutive internet daily dairy study and finally these associations were replicated in an additional preregistered daily diary study. In total, we consecutively investigated more than 200 participants with different methods and questionnaires targeting to measure a broad spectrum of creativity and creative potential indices. Our approach was motivated by the idea that although one single study might not tell us much about the link between creativity and meaningful coincidences, three studies might. Unfortunately, at the same time, a higher number of studies also leaves us with a higher number of open questions.

Although the experience of meaningful coincidences was linked to creative activities and creative achievements, follow-up analyses indicate a less coherent pattern of findings. For creative activities, literature, sports, and performing arts (at a trend) might be responsible for the significant relationship with meaningful coincidences throughout the three studies. For creative achievements cooking seems to show the most consistent pattern of associations. However, it is important to consider that these associations strongly depend on sample characteristics and if participants show creative achievements and creative activities in these very sub-categories. This means that while the sum score can reliably estimate participant’s creative achievements and activities, the sub-scores may not necessarily.

Thus, we should consider this report as a preliminary, first attempt to study the benevolent trajectory of meaningful coincidences indicated by the assocition with real-world creativity. Although the novel findings presented here are in accordance with the assumption of a connection between the experience of meaning and creativity (e.g., [2,10]), we still do not know much about the mechanisms providing this robust link.

#### What can we learn about meaningful coincidences?

Following from the study results, we can conclude that assessing the experience of meaningful coincidences is possible by means of an internet daily diary approach. Applying daily diaries reduces systematic recall biases, brings more ecological validity to our study results and allows to measure the dynamic of meaningful coincidences and synchronicity as well [37]. The assessment of changes from moment to moment is an important prerequisite for future research uncovering potential mechanisms responsible for the experience of meaningful coincidences. This is even more important since the experience of meaningful coincidences is a naturally occurring phenomenon, which experimenters cannot induce artificially. Until to date, research considered meaningful coincidences a (personality) trait only [2]. Since in our studies, most people perceived at least one meaningful coincidence and synchronicity during the span of a week, a more dynamic assessment by means of a daily diary or experience sampling methods might help to shed more light on this phenomenon [63,64]. Furthermore, the within-person variance of meaningful coincidences was assessed reliably (at the lower border) and was meaningfully associated with PA as well as NA (study 2). This finding was only partly replicated in study 3, yet it encourages future attempts to study the facilitating mechanisms of meaningful coincidences and synchronicity by means of these methods.

#### What can we learn about the association between meaningful coincidences and creativity?

Is the experience of meaningful coincidences related to creativity? The answer to this exciting question is complex. For one, creativity is not a single, homogeneous entity and can be understood as a potential, a personality trait, an activity, as well as an achievement. Furthermore, creativity and the experience of meaningful coincidences can be studied at two levels (between-person and within-person). The present studies did not find strong evidence suggesting that seeing connections and meaningful patterns which are *hidden from the view of others (cf*. [16]) help to overcome mental blocks, enhance thinking out of the box, or increase the chance to reach more creative ideas. Although, the association with real-life creativity seems robust, relationships with other aspects of creativity such as openness to experience or people’s creative potential are much weaker and show lower effect sizes ([18,34]). The association seems to be more about displaying creative activities such as cooking and writing blogs (as well as creative achievements) and less about the basic potential to come up with many original ideas to solve creative problems.

The reason for the obtained robust link between creative activities and meaningful coincidences is unclear. We did not find evidence for a convincing mechanism; however, PA might still represent a target for future studies. At least in study 3, we found that PA states seem to mediate the association between synchronicity and the number of creative activities in everyday life. Nevertheless, due to the study findings, we can rule out at least two reasons for the association between meaningful coincidences and creativity: First, the association between the propensity to experience meaningful coincidences and real-life creativity is not (strongly) based on a higher creative potential of these people. People who experienced more meaning in meaningless noise did not necessarily show a higher creative potential on the implemented creativity task, while at the same time, reporting higher creative achievement and more creative activities. The null finding for creative potential is in line with previous research indicating that people with a higher creative potential also have higher inhibitory and executive skills (e.g., [22,65,66]). These skills seem to somewhat oppose the experience of meaningful coincidences, who were reported to show reduced working memory capacities [67] and reduced inhibitory control [2,20] as well as reduced grey matter in relevant cortical areas [28]. In our three-study approach, we used three different measures of creative potential, a figural drawing task (TCT-DP; [68]), naming unrelated words (DAT; [45]), and finding original uses of everyday objects (AUT; [60]). Not a single index of all these measures was associated with meaningful coincidences (or synchronicity) at the between-person level. This is even more compelling since, the creative potential measures seemed to be valid and showed the expected associations with other creativity indices such as creative achievements (e.g., [35]). When taking the effect sizes into account, we can conclude that if there is any association at all, it is likely small. Therefore, future studies with larger samples have to investigate this association more deeply and should also place emphasis on possible within-person associations of meaningful coincidences and creative abilities (see e.g., [27,69]).

Second, affect might not explain the association between meaningful coincidences and creative activities. Negative affect was associated with more creative activities and meaningful coincidences, which might indicate that people with a higher NA have a higher need to give their lives a meaning [23,70]. However, the variance of this association was not shared between both variables, thus discouraging a mediating mechanism. Similarly, PA was associated with meaningful coincidences at the between-person and within-person level, which however only partly explains the link between real-life creative activities and the experience of meaningful coincidences.

A further target for future research is curiosity, described as the tendency to explore uncertain environments [71]. Following the proposed framework by Ivancovsky [71], curiosity is associated with increased creativity via novelty-seeking. This might explain why people who perceive more meaning in randomness might also engage in more creative activities and show more creative achievements (see e.g., [34] for positive schizotypy). However, this assumption needs further empirical support.

## Conclusions

Taken together, our three consecutive studies allow three main conclusions. First, our studies showed that people who experienced more meaningful coincidences are also more often engaged in real-life creativity such as creative activities and creative achievements [11]. This association at the between-person level was also found for the within-person level, which indicates the relevance of meaningful coincidences for real-life creativity and vice versa. Second, PA and NA are related to more perceived meaningful coincidences, predominantly at the level of the person. There are good reasons to assume that this link exists at the within-person level too. Nevertheless, PA and NA seem not to serve as strong mechanisms explaining the link between meaningful coincidences and creative activities. Third, creative potential seems less strongly linked with the experience of meaningful coincidences as literature might have implicitly suggested. The robustly observed association between creative activities and achievements with the perception of meaning fits the ideas of Kaufman [70], who suggested that creativity can help people find meaning in their lives. This statement may also be true for the experience of meaningful coincidences, which can be understood as a creative act in which people connect unrelated but significant events and give these events and consequently, also their lives, some meaning.

## Supporting information

S1 Table Pearson correlations between coincidences and the subscales of the ICAA for study 1, study 2, and study 3. *p* values in parentheses. Creative achievements = CAch, Creative activities = CAct.
(DOCX)
