# Supplementary material for: Experiencing more meaningful coincidences is associated with more real-life creativity? Insights from three empirical studies
Source: PLoS One. 2024 May 24;19(5):e0300121. doi: 10.1371/journal.pone.0300121 (PMC11125470; doi:10.1371/journal.pone.0300121)
Supplement: S1 Table — p values in parentheses. Creative achievements = CAch, Creative activities = CAct. (DOCX) [file pone.0300121.s001.docx]

S1 Table 1. Pearson correlations between coincidences and the subscales of the ICAA for study 1, study 2, and study 3

|  | Study 1 (n=69) | | Study 2 (n=93) | | Study 3 (n=80) | |
| --- | --- | --- | --- | --- | --- | --- |
|  | Coin-CAct | Coin-CAch | Coin-CAct | Coin-CAch | Coin-CAct | Coin-CAch |
| Sum-score | **.352 (.003)** | .165 (.174) | **.345 (.001)** | **.306 (.003)** | **.307 (.006)** | **.226 (.044)** |
| Literature | **.243 (.045)** | .129 (.291) | **.301 (.003)** | .181 (.082) | **.346 (.002)** | .087 (.442) |
| Music | .081 (.511) | .123 (.314) | **.319 (.002)** | **.414 (.001)** | **.236 (.035)** | .191 (.090) |
| Arts and crafts | **.276 (.022)** | .037 (.764) | **.228 (.028)** | .164 (.115) | -.017 (.879) | .037 (.746) |
| Cooking | **.353 (.003)** | .210 (.083) | .160 (.127) | **.220 (.034)** | .064 (.572) | **.228 (.042)** |
| Sports | **.273 (.023)** | -.013 (.915) | **.286 (.005)** | **.266 (.010)** | **.228 (.042)** | .109 (.337) |
| Visual arts | **.244 (.043)** | -.096 (.434) | .078 (.456) | .022 (.835) | **.318 (.004)** | .213 (.058) |
| Performing arts | **.278 (.021)** | .203 (.094) | .174 (.095) | **.261 (.012)** | **.284 (.011)** | .210 (.062) |
| Science and engineering | .100 (.413) | .112 (.358) | .172 (.100) | -.055 (.603) | .127 (.260) | .061 (.592) |

Note. *p* values in parentheses. Creative achievements =CAch, Creative activities = CAct.
